# Bony Landmarks for Determining the Mechanical Axis of the Femur in the Sagittal Plane during Total Knee Arthroplasty

**DOI:** 10.4055/cios.2009.1.3.128

**Published:** 2009-08-17

**Authors:** Jai-Gon Seo, Byung-Kuk Kim, Young-Wan Moon, Jong-Hyun Kim, Byeong-Ho Yoon, Tae-Keun Ahn, Dong-Hoon Lee

**Affiliations:** Department of Orthopedic Surgery, CHA Bundang Medical Center, CHA University, Seongnam, Korea.; * Department of Orthopedic Surgery, Samsung Medical Center, Sungkyunkwan University School of Medicine, Seoul, Korea.; † Department of Orthopedic Surgery, CHA Gumi Medical Center, CHA University, Gumi, Korea.

**Keywords:** Bony landmark, Sagittal alignment, Mechanical axis, Total knee arthroplasty

## Abstract

**Background:**

There is no accepted landmark for the mechanical axis of the femoral axis in sagittal plane in conventional total knee arthroplasty.

**Methods:**

As palpable anatomic landmarks of the femur, lateral epicondyle, and anterior margin of the greater trochanter were identified. The line connecting these two landmarks was defined as the "palpable sagittal axis". The mechanical axis of the femur was compared with the palpable sagittal axis and the distal femoral anterior cortex axis. These axes were also compared with sagittal bowing of the femur.

**Results:**

The distal femoral anterior cortex axis and the palpable sagittal axis were flexed by 4.1° and 2.4° more than the sagittal mechanical axes, respectively (*p* < 0.05). However, the palpable sagittal axis was not correlated with sagittal bowing of the femur (Spearman's rs, 0.17; *p* = 0.14).

**Conclusions:**

The palpable sagittal axis showed a consistent relationship with the sagittal mechanical femoral axes regardless of the severity of the sagittal bowing of the femur.

Many variables, including patient factors and surgeon's skill, can impact upon prosthetic longevity and patient outcome.1) Nevertheless, the factor of greatest importance during total knee arthroplasty (TKA) is prosthetic alignment, and in particular, femoral component alignment is crucial to function and long-term survival.^1^-4) In contrast to computer-navigated TKA systems, where movements of hips, knees, and ankles are analyzed by mathematical algorithms to determine mechanical leg alignment, including the sagittal mechanical axis, there is no accepted landmark for the sagittal femoral axis in conventional TKA. If the mechanical axis of the femur in the sagittal plane can be obtained during conventional TKA, it can enhance the extramedullary femoral alignment system without navigation equipment and can modulate the sagittal position of the femoral component in the case of excessive femoral bowing. In the present study, the possibility of using palpable landmarks to determine the sagittal mechanical axis of the femur was investigated.

## METHODS

**Fig. 1:**
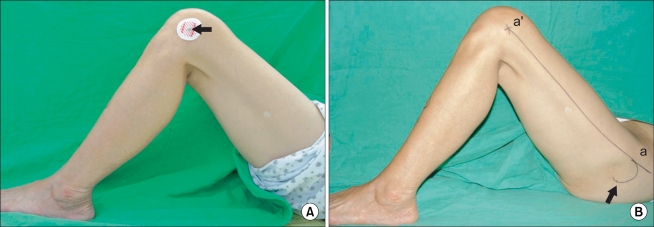
(A) Lateral epicondyle of the femur was marked with an electrocardiogram lead (arrow). (B) Two kinds of palpable anatomical landmarks - anterior margin (a) of the greater trochanter (arrow) and lateral epicondyle of the femur (a') - were identified and the line aa' was defined as the "palpable mechanical axis" of the femur in the sagittal plane.

**Fig. 2:**
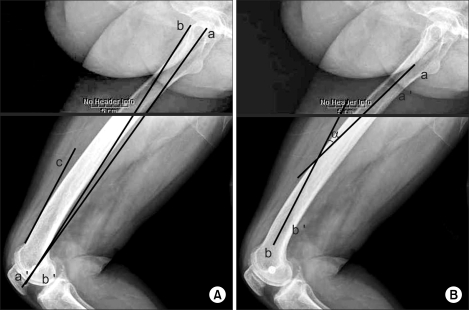
(A) The mechanical axis of the femur in the sagittal plane was defined as the line connecting the center of the femoral head (a) and the center of the condyles (a'). The line connecting anterior margin of the greater trochanter (b) and the center of the lateral epicondyle of the femur (b') was defi ned as the "palpable mechanical axis." The axis of the anterior cortex of the distal femur (c) was defined as a line along the cortex just above the femoral condyles. (B) Sagittal bowing of the distal femur as the angle (α) between the longitudinal axis of the proximal and distal femur. The proximal axis was obtained connecting the centers of the medullary canal at the level of the lesser trochanter (a) and the point (a') 4 cm distal to the lesser trochanter. The distal axis was obtained connecting the centers of the medullary canal at the junction (b) between the femoral shaft and the condylar region and the point (b') 4 cm proximal to it.

We conducted this prospective, observational study of 76 consecutive primary posterior cruciate ligament-substituting TKAs (Scorpioe®, Osteonics, Stryker, NJ, USA) in 76 patients. Six men and 70 women were enrolled, and the mean patient age at the time of surgery was 69 ± 6.6 years (range, 54 to 82 years). Seventy-five patients had a confirmed diagnosis of degenerative osteoarthritis, and one patient had osteonecrosis of a distal femur. Two types of palpable anatomic landmarks, i.e., the lateral femoral epicondyle and the anterior margin of the greater trochanter, were identified preoperatively, and the lateral femoral epicondyle was marked with an EKG lead (Fig. 1). Lateral plane radiographs of the entire femurs were obtained with complete overlapping of the femoral condyles (Fig. 2). The axis of the distal femoral anterior cortex was defined as a line along the cortex just proximal to the femoral condyles (Fig. 2A). The axis of the distal femoral anterior cortex is important, especially in an extramedullary femoral alignment system because total knee systems that employ an extramedullary alignment guide are actually using the anterior surface of the distal femur as a reference for the sagittal positioning of the femoral component. We also obtained the axis by joining the center of the lateral epicondyle of the femur and anterior margin of the greater trochanter, and defined this as the "palpable sagittal axis" (Fig. 2A). The palpable sagittal axis is a line that the surgeon can obtain intraoperatively about the sagittal axis of the femur without the aid of radiographic devices. The sagittal mechanical axis was arbitrarily defi ned as the line connecting the center of the femoral head and the center of the lateral femoral condyle. A TKA will be ideal if the implant is positioned tangential to the mechanical axis both in the coronal and sagittal axes. The differences between these three axes were measured (Fig. 2A). We defined sagittal bowing of the distal femur as the angle between the longitudinal axis of the proximal and distal femur. The distal end was defined at the junction between the femoral shaft and the condylar region, which was indicated by a flare of the posterior cortex.5) A line connecting the centers of the medullary canal at the distal end as defined, and the point 4 cm proximal to the end, was obtained. The proximal end was defined at the level of the lesser trochanter5) and a line connecting the centers of the medullary canal at the proximal end, and the point 4 cm distal to the end was obtained. The angle between the lines was defined as sagittal bowing (Fig. 2B). We also analyzed the correlations between the axis of the distal femoral anterior cortex and the sagittal mechanical axis, and between the palpable sagittal axis and the sagittal mechanical axis.

All measurements were performed using Picture Archiving Communication System (PACS, General Electric, Milwaukee, WI, USA). Statistical analysis was performed using SPSS ver. 11.5 (SPSS Inc., Chicago, IL, USA). The t-test for paired samples was used to compare axes, and correlations between variables were determined using Pearson's correlation. *p*-values < 0.05 were regarded as significant.

## RESULTS

The axis of the distal femoral anterior cortex was 4.1° (range, 1.5 to 11.7°; SD, 2.8°) more flexed than the sagittal mechanical axis (*p* < 0.05), and the palpable sagittal axis was 2.4° (range, 0.4 to 4.2°; SD, 0.9°) more flexed than the sagittal mechanical axis (*p* < 0.05). The mean sagittal bowing was 13.9° (range, 6.2 to 24.5°; SD, 4.2°), and the axis of the distal femoral anterior axis was significantly correlated with sagittal bowing of the femur (Spearman's rs, 0.73; *p* < 0.0001). However, the palpable sagittal axis was not correlated with sagittal bowing of the femur (Spearman's rs, 0.17; *p* = 0.14).

## DISCUSSION

Many authors have concluded that correct alignment of the lower extremity is correlated with clinical success in TKA.^2^,4) Moreover, improper positioning of components during surgery, particularly in the coronal plane, may lead to accelerated wear and failure.2) However, the majority of studies have focused on coronal alignment in TKA; sagittal alignment has not been given the attention in the literature it deserves. Although femoral component alignment has been thoroughly studied,^2^,^3^,^6^-8) the importance of femoral sagittal bowing in TKA has not been widely studied. Sagittal bowing of the femur should be considered in TKA because the axis of the distal femur is more flexed than the sagittal femoral mechanical axis as sagittal bowing increases.

The human femur is usually described as being sagittally curved and convex anteriorly.^9^-11) In an extensive study on femoral sagittal curvature, Walensky11) identified racial differences. Specifically, it was shown that American-Indians exhibited greater anterior curvature than Caucasians and African-Americans, and that the femora of Eskimos are more closely related to those of American-Indians. Tang et al.5) demonstrated that the distal one-third of the femora is significantly more bowed than the middle and proximal portions. This excessive sagittal bowing of the distal femur implies that the axis of the distal femoral anterior axis is more flexed than the sagittal mechanical axis. Theoretically, the femoral component should reconstruct the lateral contour of the distal femur, but this cannot be true at higher levels of femoral sagittal bowing, especially in east Asians.5) If the femoral component is too flexed, the anterior surface of the femoral cam box is more likely to impinge on the anterior surface of the polyethylene post when the knee comes into full extension, which causes accelerated wear of the post.^12^,13) Tang et al.5) recommended that the size of the entry hole of the femur should be limited to prevent the intramedullary rod from tilting in those patients with obvious femoral bowing. When using image-free navigation systems, femoral sagittal mechanical axes are determined using localizers constructed with infrared diodes and by mobilizing hip, knee, and ankle joints.14) The movements of localizers are analyzed using mathematical algorithms to determine individual mechanical leg alignments.14) However, femoral sagittal mechanical axes cannot be determined using the conventional TKA technique because there are no palpable landmarks. If the femoral sagittal mechanical axis can be determined in the operative field, the amount of flexion of the femoral component can be modulated when sagittal bowing is significant. The "palpable sagittal axis" defined in this study was 2.4° more flexed than the sagittal mechanical axis and showed a consistent relationship with the axis regardless of the severity of sagittal bowing. Our study enables the sagittal mechanical axis of the femur to be determined using a conventional technique. It is useful when determining the sagittal axis of the femoral component in patients with increased femoral sagittal bowing, and it can facilitate the extramedullary femoral alignment system without navigation equipment.

The sagittal mechanical axis of the femur can be defined in the operative field without navigation using palpable bony landmarks. The sagittal axis of the femoral component can be modulated in cases with excessive femoral bowing and the extramedullary femoral alignment system can be facilitated.
